# Modernisation, smoking and chronic disease: Of temporality and spatiality in global health

**DOI:** 10.1016/j.healthplace.2015.04.004

**Published:** 2016-05

**Authors:** David Reubi

**Affiliations:** Department of Social Science, Health and Medicine, King’s College London, London WC2R 2LS, United Kingdom

**Keywords:** Temporality, Spatiality, Global health, Non-communicable disease, Modernisation

## Abstract

This article explores the spatio-temporal logics at work in global health. Influenced by ideas of time–space compression, the global health literature argues that the world is characterised by a convergence of disease patterns and biomedical knowledge. While not denying the influence of these temporalities and spatialities of globalisation within the global health and chronic disease field, the article argues that they sit alongside other, often-conflicting notions of time and space. To do so, it explores the spatio-temporal logics that underpin a highly influential epidemiological model of the smoking epidemic. Unlike the temporalities and spatialities of sameness described in much of the global health literature, the article shows that this model is articulated around temporalities and spatialities of difference. This is not the difference celebrated by postmoderns, but the difference of modernisation theorists built around nations, sequential stages and progress. Indeed, the model, in stark contrast to the ‘one world, one time, one health’ globalisation mantra, divides the world into nation–states and orders them along epidemiological, geographical and development lines.

## 1 Introduction

Influenced by the theories on globalisation that became so pervasive after the end of the Cold War, many of those who have written on global health assume that the last decades of the twentieth century have been marked by an accelerated compression of time and space (e.g. Beaglehole and McMichael, 1999; Walt, 2000). For them, the world has become a global village characterised by political, economic, and social integration as well as temporal simultaneity. This, they contend, is the consequence of trade liberalisation policies and technological innovations like air travel and the Internet, which have brought about growing flows of people, knowledge, capital and goods around the world. Applying these ideas to public health and biomedicine, these commentators explain that the world we now live in is characterised by a convergence of disease patterns, biomedical knowledge and public health strategies. Often these arguments have been made in relation to infectious diseases, as with the idea that air travel has allowed for the rapid spread of microbes around the global (e.g. Garrett, 1995; Youde, 2012). More importantly for us, similar ideas have also been articulated about non-communicable diseases (NCDs) and their risk factors and, specifically, the smoking epidemic and the chronic diseases it contributes to (e.g. Yach and Bettcher, 1999; Lee, 2003). So, for example, many commentators have argued that smoking and lung cancer are a global epidemic caused by trade liberalisation and multinational tobacco companies. Likewise, others have argued that ‘global advocacy’ in the field of tobacco control was made possible by the Internet, which allowed activists from around the world to ‘interact simultaneously’ (e.g. Yach and Bettcher, 2000; Lee and Collin, 2005).

There is little doubt that these temporalities and spaces of globalisation shape many theories, practices and materialities in today's global health and chronic disease complex (McGoey et al., 2011). But, as an emerging body of research suggests, there are other, often-conflicting spatio-temporal logics at work within this complex (e.g. Tousignant, 2013; Beisel, 2014; cf. also Lakoff and Collier, 2008; Fassin, 2012; Anderson, 2014). This article contributes to this research by arguing that there exists, within the contemporary field of global tobacco control, what I term temporalities and spaces of modernisation that have been extremely influential and stand in stark contrast to the spatial and temporal logics of globalisation. To do so, I examine a statistical model of the global smoking epidemic that has shaped the way tobacco control advocates have thought for the last twenty years and which was elaborated by epidemiologist Alan Lopez and his colleagues at the World Health Organisation (WHO) in the early 1990s. Specifically, drawing on extensive archival and ethnographic research on the international tobacco control movement,^1^ I unpack how this model links the different temporal phases of the epidemic with particular disease patterns, public health policies, geographical regions and levels of development. I also show how many of its assumptions can be traced back to postwar modernisation and development theories. I conclude by exploring what this might mean for our understanding of global health. But, before doing so, I examine the temporalities and spaces of globalisation that can be found in much of the literature on global health.

## 2 Temporalities and spatialities of globalisation

In *The Condition of Postmodernity*, geographer David Harvey argued that the world was experiencing a ‘time–space compression’:

As space appears to shrink to a ‘global village’ of telecommunications … and as time horizons shorten to the point where the present is all there is … so we have to learn to cope with an overwhelming sense of *compression* of our spatial and temporal worlds (Harvey, 1989, p. 240).

He further observed that this time–space compression, which had been on-going since at least the mid-nineteenth century, had recently accelerated because of radical changes in the nature of capitalism and revolutions in transport and communication technologies. While particularly influential, Harvey was certainly not alone in articulating these ideas. Indeed, the last decades of the twentieth century saw a growing number of publications and debates on this topic, so much so that ideas about time–space compression and globalisation more generally had gained widespread acceptance by the late 1990s (May and Thrift, 2001, Scholte, 2005).

It is therefore no surprise that these ideas have been so influential among many of those writing on global health over the last fifteen years (e.g. Walt, 2000; Lee and Collin, 2005).^2^ Borrowing from the work of Marshall McLuhan, David Harvey, Anthony Giddens and others, these writers imagine that the post-Cold War period has been marked by ‘a process of increasing economic, political and social interdependence and global integration’ (Yach and Bettcher, 1998, p. 735). ‘Time and space’, they feel, is ‘collapsing’ (Yach and Bettcher, 2000, p. 206). The world is becoming a ‘global village’ (Lee and Collin, 2005, p. 15) with ‘a sense of transworld simultaneity and instantaneity’ (Lee, 2003, p. 105) and a ‘shared cosmopolitan culture’ (Yach and Bettcher, 2000, p. 206). Following the literature on globalisation, these writers view this ‘process of closer integration’ as being the result of two key factors (Walt, 2000, p. 1). The first is ‘neoliberalism’ and, specifically, ‘trade liberalisation’ (Lee, 2003, p. 65; Harman, 2012, p. 5). The second is the ‘revolution in communications and transportation technologies’ from the Internet to the aeroplane (Daulaire, 1999, p. 22). These factors, they believe, enable the ever growing ‘flows of information, goods, capital and people across political and geographical boundaries’ that bring about a global convergence of social, political and economical life (Daulaire, 1999, p. 22).

What is innovative in these writings on global health is the way they conceive public health and biomedicine through the lense of globalisation and time–space compression (Brown et al., 2006, Fassin, 2012). Thus, for these writers, the world is ‘a global health village’ characterised by a convergence of disease patterns, biomedical expertise and public health interventions (Yach and Bettcher, 2000, p. 736). Often, these arguments are made in relation to infectious diseases (e.g. Garrett, 1995; Weinberg, 2005; Youde, 2012). Many of these writers argue, for example, that the development of air travel has led to ‘the microbial unification of the world’ by allowing for the rapid spread of pathogenic microorganisms (Berlinguer, 1999, p. 18). Another illustration is the way in which the development of new Internet-based, epidemiological surveillance systems allow public health authorities across the globe to know about and prepare against pandemics ‘in real-time’ as they unfold (e.g. Weir and Mykhalovskiy, 2010; Caduff, 2014).

Importantly for us, many commentators writing on global health have applied ideas about globalisation and time–space compression to their analysis of NCDs and their risk factors. Some of them have written on the relationship between trade liberalisation in the food industry and the rise of unhealthy diets and NCDs like diabetes (e.g. Smith, 2003; Chopra, 2005). Others have explored the impact of globalisation on the alcohol industry and the chronic disease burden (e.g. Gilmore, 2009; Collin et al., 2014). But, most of these commentators have focused on smoking (e.g. Yach and Bettcher, 1999; Lee, 2003; Collin, 2005). The reasons for this are mainly historical: smoking was the first NCD risk factor to be addressed in global health with the adoption of the *WHO Framework Convention on Tobacco Control* (*FCTC*) in the early 2000s and now serves as a model for tackling other key NCD risk factors (Yach et al., 2003, WHO, 2003, Casswell and Thamarangsi, 2009). For these commentators, smoking is conceived as a ‘global epidemic’ defined by worldwide mortality and morbidity figures. One author, for example, argues that ‘the global tobacco epidemic’ kills an ‘estimated four million people’ per year around the world (Collin, 2003). ‘Transnational tobacco companies’, they suggest, are the main driver of this epidemic (Collin, 2005, p. 114). Taking advantage of recent trade liberalisation efforts, these companies are expanding their markets around the globe through sophisticated advertising and marketing campaigns purporting to spread a ‘shared [smoking] culture’ articulated around ‘global [cigarette] brands’ and the notion of ‘the global smoker’ (Yach and Bettcher, 1999, Collin, 2003). These different commentators also draw on ideas about globalisation and time–space compression to rethink the public health strategies deployed to stop the smoking epidemic. To illustrate, some argue that in order to ‘impact tobacco consumption throughout the world’ one needs ‘global norms and legal instruments’ such as the *FCTC* (Yach and Bettcher, 1998, p. 740; Harman, 2012, p. 38). Similarly, others comment that new communication technologies like the Internet have ‘profoundly improved’ the ‘prospects for global advocacy’ by allowing experts and activists everywhere to ‘interact simultaneously’ (Yach and Bettcher, 1998, Yach and Bettcher, 2000).

## 3 Contextualising the Lopez model

There is no doubt that these temporalities and spatialities of globalisation play an important role within today's global health field, but they are certainly not the only concepts of time and space to shape this field. The temporal and spatial forms associated with an influential epidemiological model of the smoking epidemic elaborated by Alan Lopez and others are a good example of such alternative concepts of time and space.^3^ But, before we look at these forms in more detail, it is important we discuss the context in which the model was developed. Lopez's model – which outlines how the smoking epidemic develops in a population over time by charting changes in prevalence, mortality, public attitudes and policies – was designed in the early 1990s by three WHO experts: Lopez, an Australian demographer who worked as chief epidemiologist for the Geneva-based organisation's *Tobacco or Health Programme* (*THP*); Neil Collishaw, a sociologist and long-term anti-smoking advocate from Canada also based at the *THP*; and Tapani Piha, a specialist in community medicine from Finland who worked on tobacco control for the WHO's Regional Office for Europe (WHO Europe). The three men outlined their epidemiological model in an article entitled ‘A Descriptive Model of the Cigarette Epidemic in Developed Countries’, which appeared in 1994 in *Tobacco Control* – the leading journal in the field of international tobacco control (Lopez et al., 1994).

The Lopez model was part of growing international efforts to address the smoking epidemic at the time (Reubi and Berridge, forthcoming). The internationalisation of tobacco control has a long history stretching back to the 1960s and the organisation of the World Conferences on Tobacco or Health every few years. But, it is really from the 1980s onwards that international efforts in the field really picked up. One important initiative, though probably not as influential as the International Union against Cancer's *Smoking and Cancer Programme*, was the *THP* where Lopez and Collishaw devised their model. As the WHO's first permanent programme on tobacco control, the *THP* was an understaffed and underfunded operation that, among others, developed standardisation protocols for smoking prevalence surveys and carried out capacity building workshops in the developing world (Chollat-Traquet, 1990). Another similar initiative, albeit a more regional one, was the *Action Plan for a Tobacco-Free Europe* launched by WHO Europe and for which Piha was a consultant (WHO Europe, 1993). An important aspect of international tobacco control efforts during this period was the problematisation of smoking in what was then called ‘the Third World’ (Reubi, 2013). Up to the 1980s, experts thought that chronic diseases and contributing risk factors like smoking were exclusive to the rich, industrialised nations of the North and that the developing world was all about infectious diseases, malnutrition and poverty. The publication of the WHO report on *Smoking Control in Developing Countries* in 1983 marked a shift in thinking (WHO, 1983). Increasingly, there was a recognition that the smoking epidemic was spreading to the Third World. This, it was imagined, was the result of raising disposable incomes associated with successful economic development and modernisation as well as the tobacco industry's efforts to create new markets throughout Latin America, Asia and Africa. It was to prevent developing countries facing the additional burden of smoking-related diseases that international initiatives like the *THP* sought to build tobacco control capacity in the Third World.

The Lopez model was also part of late-twentieth century efforts by epidemiologists to formulate credible, global estimates for smoking prevalence and smoking-attributable mortality. At the time, there were a lot of doubts about the reliability of the numbers on smoking produced by the WHO. As an expert involved in international tobacco control efforts during this period remembered:

We always questioned smoking statistics in those days. Especially those from the WHO, which we thought must simply be wrong.

These doubts were not limited to the data about smoking prevalence and smoking-attributable mortality but extended to the global mortality estimates for most diseases and risk factors published by the WHO (Smith, 2013). The reasons for these doubts were multiple. First, in many countries – indeed, most developing countries – there were no nation-wide surveys on smoking habits and no national death registers from which to draw data on tobacco-related mortality (Mackay and Crofton, 1996). Second, the epidemiological models to estimate smoking prevalence and smoking-attributable mortality when data was missing were often very crude (WHO, 1990). Third, there were many instances of double-counting within WHO, with all departments attributing deaths to the diseases for which they were responsible to increase their funding (Smith, 2013). The increasing numbers of epidemiological investigations that were conducted around that time were often framed as a solution to these problems of reliability. Alan Lopez himself was closely associated with two of these investigations, which directly fed into and shaped the model of the smoking epidemic he developed with Collishaw and Piha. The first one was a research project led by Oxford epidemiologist Richard Peto that computed more reliable, global figures for smoking-attributable mortality using a novel estimation technique (Peto and Lopez, 1990, Peto et al., 1994). The second investigation was the Global Burden of Disease project led by Christopher Murray at Harvard that sought to establish global estimates of mortality and disability to allow for more rational policymaking (Murray and Lopez, 1996).

The Lopez model has been and continues to be hugely influential in the field of global tobacco control. The article in *Tobacco Control* where the model was outlined in 1994 has been cited more than 850 times according to Google Scholar. Unsurprisingly, most experts and activists in the field know and regularly refer to it. As two high-profile tobacco control experts observed:

The Lopez model has been incredibly influential … It is still used as a frame of reference today even though it was published 20 years ago.

[The Lopez model] is still as valid today as it was twenty years ago. I still very much use it my work. For all its potential problems, it generally fits what is going on.

The importance of the model in shaping the thinking of tobacco control advocates around the globe was also recently recognised by the editors of the leading journal in the field: *Tobacco Control*. The journal's 20-year anniversary issue, which sought to review ‘the major achievements’ in the field, contained one article by Peto, Lopez and others in which they revisited and, aside from a change to smoking and smoking-attributable mortality rates for women, confirmed the overall validity of their 1994 ‘landmark model’ in the light of new epidemiological data (Malone and Warner, 2012, pp. 72–73; Thun et al., 2012). The model has also been very influential for advocacy and policymaking. Indeed, both tobacco control experts and historians agree that, together with the new epidemiological estimates provided by Peto and his colleagues, it played a decisive role in the adoption of the *FCTC* in the early 2000s (Reynolds and Tansey, 2012). More recently, experts mandated by the Bill and Melinda Gates Foundation used the Lopez model to show the surprisingly large number of lives that could be saved through the prevention of the upcoming smoking epidemic in sub-Saharan Africa, legitimising thereby the philanthropy's efforts to develop a tobacco control movement across the subcontinent (Blecher and Ross, 2014).

## 4 Of time and space in the Lopez model

To unpack the notions of time and space that underpin the Lopez model, one needs to examine the way the model imagines the smoking epidemic. As already mentioned, the model outlines how the epidemic develops in the population of a country over a hundred-year time period. The model is based on statistical data from a few Western countries and, in particular, the USA and the UK, which were among the first to experience the epidemic and where there has been the necessary epidemiological infrastructure to record smoking prevalence and smoking-related mortality (Berridge, 2007, Brandt, 2007). Many of the assumptions around which the Lopez model is built, such as the hundred-year period over which the epidemic is represented, derive from the particularities of this data. The model identifies four successive, 25-year long phases – which Lopez and his colleagues term Stage I, Stage II, Stage III and Stage IV – through which the epidemic unfolds. For all four stages, the epidemic is characterised along three explicit variables. The first is smoking prevalence, understood as ‘the percentage of the adult population who smoke regularly’ (Lopez et al., 1994, p. 243). The second is smoking-related mortality, defined as the ‘numbers of deaths’ caused by smoking ‘through a variety of diseases, principally several sites of cancer, major vascular diseases and chronic lung diseases’ (Lopez et al., 1994, p. 243). Given the difference in smoking patterns among men and women in the USA and UK, the model further breaks down these first two variables by male and female. The third variable, which unlike the first ones is not numerical, is public attitudes to smoking and the state of tobacco control policies.

**Fig. 1:**
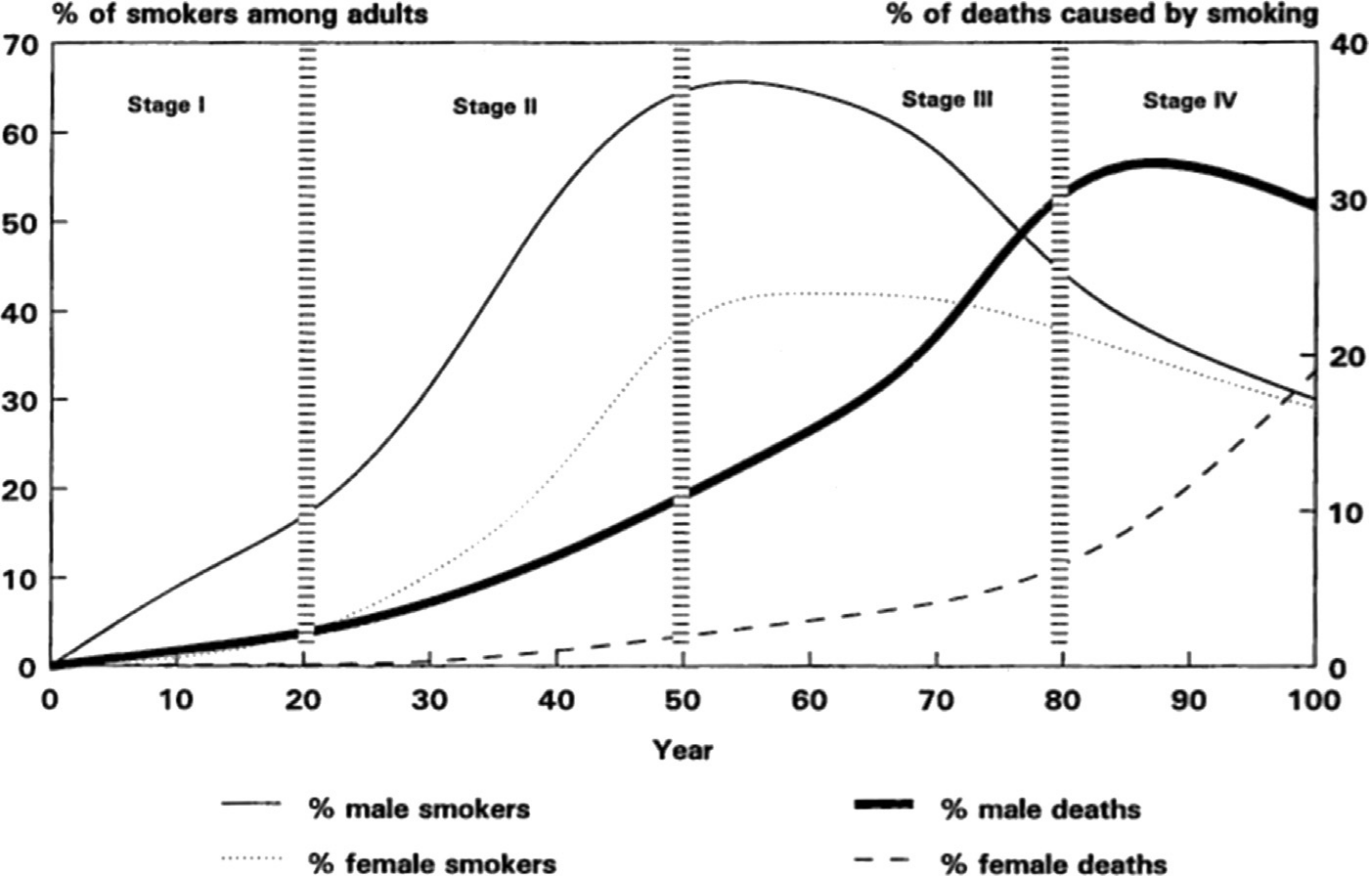
The model of the smoking epidemic as found in Lopez’ 1994 *Tobacco Control* article.

Lopez and his colleagues offer both a narrative and a graphic account of the four stages of the epidemic using these three variables (cf. Fig. 1). Stage I describes the beginning of the smoking epidemic: male prevalence starts rising reaching 15%, while female prevalence remains low ‘because of socio-cultural factors’ which discourage women from smoking; ‘death and disease due to smoking are not yet evident’; and ‘smoking becomes socially acceptable and tobacco control strategies remain underdeveloped, with priority being given to reducing malnutrition and the burden of infectious diseases’ (Lopez et al., 1994, p. 244). Stage II sees the epidemic develop further: male prevalence continues to grow rapidly, peaking at 60%; female prevalence increases dramatically to reach over 30%; smoking-related deaths among men start rising, typically mirroring the rise in smoking prevalence with a twenty-year time-lag because of the late onset of lung cancer, chronic obstructive pulmonary disease and some cardio-vascular diseases; and tobacco control policies remain weak, with ‘a lack of public and political support, in part because of the risks of tobacco use may still not be widely understood’ (Lopez et al., 1994, p. 244).

Stage III seems to represents a turning point in the epidemic: ‘male prevalence begins to decline’ to around 40% while female prevalence plateaus at 40% before decreasing; smoking-related mortality among men rises dramatically, accounting for 30% of all deaths by the close of the period; smoking-attributable mortality among women also starts to grow; at the same time, public attitudes to smoking change, with ‘knowledge about the health hazards of tobacco [now] generally widespread’ and smoking becoming a ‘socially abnormal behaviour’ leading to the implementation of tobacco control policies (Lopez et al., 1994, pp. 244–245). Stage IV represents the tail end of the epidemic: ‘smoking prevalence for both sexes continues to decline’; smoking-related mortality among men begins to slowly decrease, while mortality among women is still increasing, reaching 20% by the end of the stage; and public attitudes towards smoking harden and anti-smoking policies become more comprehensive (Lopez et al., 1994, p. 245).

Crucially for the argument made here, there is a fourth, unspoken variable along which Lopez and his colleagues characterise the smoking epidemic: the level of economic development of the country in which the epidemic is unfolding. Indeed, they implicitly posit that the more developed a country is, the more it will have progressed through the stages of the smoking epidemic. Moreover, they tacitly associate the level of development and stages of the epidemic with different geographical regions. This is evident in the way Lopez and his colleagues relate particular stages of the epidemic to particular countries. Thus, they explain that ‘many developing countries, primary in sub-Saharan Africa, are currently in Stage I’, which is characterised by growing smoking prevalence rates among men and no tobacco control policies because of the prioritisation of malnutrition and infectious diseases by governments and health experts (Lopez et al., 1994, p. 246). Similarly, they assert that countries that are further along in their economic development ‘such as China, Japan and other countries of Asia, Latin America and North Africa’ are in Stage II, which is typified by dramatic increases in smoking prevalence, a lack of awareness about the dangers of tobacco and weak tobacco control policies (Lopez et al., 1994, p. 246). In contrast, they argue that most of the rich, industrialised ‘countries of Western Europe, along with Australia, Canada and the US, are nearing the end of Stage III or [have passed] into Stage IV’, which are marked by a decline in smoking prevalence, public opposition to smoking and comprehensive tobacco control policies (Lopez et al., 1994, pp. 245–246).

By linking smoking with development and geography, Lopez and his colleagues were tapping into ideas about tobacco in the developing world that had become common among public health experts after the early 1980s, when smoking was being identified as a problem for the Third World (Reubi, 2013). One such idea was the notion that developed and developing countries were at opposite ends of the smoking epidemic (e.g. WHO, 1983; Peto et al., 1994). In the former, it was thought, the epidemic was subsiding, while in the latter, the epidemic was only just beginning. As a prominent tobacco control expert argued:

The difference between developed and developing countries is one of timing … A lot of what is going on right now in the developing world is not dissimilar to what went on in the developed world, many, many decades ago.

Closely related to this first idea was the view that ‘smoke-and-health consciousness in [developing] societies lags behind that of the developed world by two or three decades’ (Warner, 1984, p. 37). As one academic with an extensive experience of the global tobacco control field noted:

In many developing countries, you do not see any support for tobacco control at any level – no governmental policies, no governmental agencies, no anti-smoking movement, no research. There is just not much of anything.

Another important idea about smoking and development that dominated the global tobacco control field after the 1980s was the notion that the two major causes of the tobacco epidemic in the Third World were: (1) the successful economic development of countries in the global South, which meant that their citizens were getting richer and had more spending power for discretionary items like cigarettes; and (2) the tobacco industry's expansion into the developing world to take advantage of this new spending power and compensate their declining sales of cigarettes in the North (e.g. WHO, 1983; Stebbins, 1990; Warner, 2000). Unsurprisingly perhaps, many international tobacco control advocates bemoaned the relationship between successful economic development and increasing smoking rates, describing it as ‘defective modernisation’ and an illustration of ‘the Third World modernising all too quickly’ (Warner, 1984, p. 37; Stebbins, 1990, p. 228).

The Lopez model did not just reiterate these pre-existing ideas about smoking and development, it elaborated them further. To start with, the model made it clear that not all developing countries were the same in terms of smoking patterns, public attitudes to smoking and tobacco control policies. As mentioned above, it tacitly assumed that countries that were more developed were at a more advanced stage of the tobacco epidemic. And it correlated these differences in smoking and development to particular geographical regions, with Asian and Latin American nations associated with higher levels of development, greater smoking prevalence and superior tobacco control programmes than African societies. As one international activist observed:

The model shows that you cannot lump all developing countries together … there are Asian and Latin American countries that one might still refer to as developing countries where there is heavy smoking by males, very low smoking by females … there are African countries where smoking is still relatively novel, where smoking is still very much on the rise and very much at the beginning of the epidemic.

Furthermore, the Lopez model also bestowed many of these pre-existing ideas about development and smoking with scientific legitimacy. It did so by translating these ideas into numbers and graphs and by having them published in an internationally recognised academic journal (Latour, 1990, Porter, 1995). This was important for global tobacco control activists, who had come to view scientific truth as a marker of their moral integrity and an advantage they had over the tobacco industry (Larsen, 2008).

It is important to note that the Lopez model's relationship with developed countries is not the same as with developing ones. For the former, the model is ‘historical in nature’ in the sense that they are currently in Stage IV after having effectively gone through the previous three stages (Lopez et al., 1994, p. 245). In contrast, for the latter, the model is more dynamic. Indeed, while these countries are currently located in Stage I or Stage II, their future is still indeterminate to a certain extent. One prospect is to do like developed societies have done before and go through the next 2 or 3 stages of the epidemic as described in the Lopez model. That means letting the epidemic follow its course before tackling it in Stage III and Stage IV. But, as Lopez and his colleagues point out, developing countries ‘can prevent history repeating itself’ and chose for themselves a better, healthier future (Lopez et al., 1994, p. 245). This necessitates them taking ‘strong public health measures to arrest the growth of tobacco consumption’ at ‘earlier stages of the epidemic’ and, specifically, ‘during Stage I or Stage II’ (Lopez et al., 1994, pp. 245–246). This, of course, was possible because of the extensive knowledge about smoking and how to tackle it accumulated by experts in the global North. As Lopez and his colleagues explained, developing countries ‘have the advantage of knowing the serious health consequences of smoking’ and have at their disposal an array of already existing, ‘effective prevention interventions’ to address the problem (Lopez et al., 1994, p. 245).

Intriguingly, Lopez and his colleagues use the case of Singapore – the poster child of successful economic development and modernisation – to illustrate their point (Reubi, 2010). As they explain, in the 1970s, the Southeast Asian Republic was both at the start of its development effort and ‘in the early part of Stage II of the cigarette epidemic’, with smoking prevalence of about 42% among men and 10% among women and weak tobacco control policies (Lopez et al., 1994, p. 246). Twenty years later, in the 1990s, at a time when Singapore was close to becoming a developed nation, it should have, if it had let the epidemic run its course unchecked, been entering Stage III, with smoking prevalence at 60% among men and 40% among women and still weak anti-smoking policies. Instead, it had, in the 1970s, adopted comprehensive tobacco control measures borrowed from Europe and North America, which had brought smoking prevalence rates down to 33% among men and 3% among women – figures that were more akin to the end of Stage IV than the start of Stage III. In other words, at the same time that the Asian city–state was going through an accelerated process of modernisation, it had also achieved to progress through Stages II and III of the smoking epidemic at about two to three times the ‘normal’ speed.

## 5 Temporalities and spaces of modernisation

Many of the spatio-temporal notions that underpin the work of Lopez and his colleagues can be traced back to the modernisation and development theories that dominated the field of international politics after World War II (Escobar, 1995, Ekbladh, 2011). As Wahlberg (2007) shows, these theories are built around a distinctive conception of human progress centred on nation–states and stages of development. For proponents of these theories, all states and their populations go through the same sequence of stages as they progress from underdevelopment to development. Modelled on the history of the rich, industrialised nations of North America and Europe, these stages are characterised by particular social, political, economic, demographic and epidemiological conditions. As Wahlberg (2007) further shows, there were many examples of this way of conceptualising and grading progress in the development literature. An influential one was the model outlined by Walt Rostow in *The Stages of Economic Growth*. At one end of the development continuum was ‘traditional society’, which, Rostow thought, was characterised by ‘pre-Newtonian science and technology’, ‘family and clan connexions’, ‘fatalism’ and a reliance on ‘agriculture’ (Rostow, 1960, pp. 4–5). At the other end was ‘the age of high mass-consumption’ typified by high ‘income’, an ‘urban’ population ‘working in offices’ and ‘durable consumer goods’ such as ‘automobiles’ (Rostow, 1960, pp. 10–11). In-between these two poles, Rostow (1960, pp. 6–10) talked about ‘take-off’ and ‘drive to maturity’, which he associated with the ‘building of an effective centralised national state’, the emergence of ‘banks and institutions for mobilising capital’, a ‘new type of enterprising men’, ‘new industries’ and an embrace of ‘modern science’.

Another influential example of this way of conceptualising progress was Abdel Omran's paper on *The Epidemiological Transition*. The distinctive aspect of Omran's work was the addition of demographic and epidemiological dimensions to the stages of development sketched by Rostow. So, according to Omran (1971, pp. 533–534), traditional society, which he termed ‘the Age of Pestilence and Famine’, was not just defined by the social, political and economic traits outlined by Rostow but also by high fertility, low life expectancy, infectious diseases and malnutrition. Likewise, Omran (1971, pp. 533–534) posited that Rostow's age of mass-consumption, which he called ‘the Age of Degenerative and Man-Made Disease’, was not only characterized by a central state, industrialisation and science but also low fertility, high life expectancy, ageing populations and chronic disease. Most of these ideas developed by Rostow and Omran could be found in what was probably the most influential postwar conceptualisation of human progress: the categories of ‘Industrialised Countries’ (First World), ‘Centrally Planned Economies’ (Second World) and ‘Middle and Low Income Countries’ (Third World) outlined by the World Bank in its first *World Development Report* (World Bank, 1978, p. ix). Indeed, the Bank associated development not only with ‘economic growth’, industrialisation, ‘improvements in transportation, communications and electric power’, a ‘dynamic entrepreneurial class’ and ‘technological sophistication’, but also with ‘a rapid expansion of education systems, growing literacy, improvements in nutrition and health conditions’, ‘greater urbanisation’ and ‘reduced fertility’ (World Bank, 1978, pp. 1–7).

These stages of development allowed experts in the field to judge the progress and development of nations by situating them in both time and space. Unsurprising, North American and European countries were deemed to be in the later stages of development and associated with progress and modernity. To illustrate, Rostow (1960, p.11) linked his age of high mass-consumption with ‘the United States’, ‘Western Europe and Japan’. In contrast, the poor, developing nations of the global South were thought to be in the earlier stages of development and viewed as traditional and backwards. In its 1978 report, for example, the Bank identified most countries in sub-Saharan Africa as Low Income while it described the majority of nations in Southeast Asia and Latin America as Middle Income (World Bank, 1978). The location of a country in time and space, however, was not immutable. Specifically, modernisation theorists thought that Third World countries could improve their levels of development and move to a more advanced stage, thus reducing the spatio-temporal gap that separated them from North America and Europe (Ekbladh, 2011). Such efforts at accelerated development were made possible by technical and financial assistance from developed countries and generally involved the establishment of a strong, centralised state together with the creation of the physical infrastructure and human resources necessary for rapid industrialisation (Reubi, 2010).

The parallels between modernisation theories and the Lopez model are readily apparent. To start with, both are articulated around the idea that all countries go through the same successive stages – stages of development for modernisation theories; stages of the smoking epidemic for the Lopez model. Furthermore, in both cases, these stages are modelled on the history of Europe and America, assuming thereby that all developing countries should follow the same path as the rich, industrialised nations of the global North. This assumption has, in the case of modernisation theories, been extensively criticised for foreclosing alternative paths of development (Escobar, 1995). It has also been problematic for the Lopez model, with, for example, women in most developing countries not taking up smoking as the model predicted (Thun et al., 2012). Another parallel between modernisation theories and the Lopez model is that they both characterise each of the stages a country goes through by an amalgam of economic, political, social, demographic and epidemiological features. Similarly, in both cases, the sequence of stages is used to position a country in time and space, making it possible to group it with some nations and differentiate it from others. Finally, both modernisation theories and the Lopez model assume that a country in the early stages of development or the smoking epidemic can, by drawing on the knowledge accumulated by experts in North America and Europe, choose to progress faster through the remaining, successive stages.

## 6 Conclusion

I have sought to draw attention to some of the temporal and spatial logics at work in the field of global health and chronic disease. As I showed, many commentators writing on global health have been strongly influenced by globalisation theories and notions of time–space compression. The world, they believe, is becoming a ‘global village’ characterised by temporal simultaneity and a convergence of political, economical and social life. This process of convergence, they also believe, is the result of the ever-growing global flows of information, goods, capital and goods across political and geographical boundaries made possible by trade liberalisation and revolutions in communication and transportation. Applying these ideas to health, they argue that the world is increasingly characterised by a global convergence of disease patterns, biomedical knowledge and public health strategies. To validate their claims, these commentators point out to a range of recent developments in global health. Some relate to infectious diseases such as the rapid spread of microbes associated with air travel and Internet-based, epidemiological surveillance systems that allow public health authorities to tackle epidemics ‘in real-time’. But many others relate to chronic diseases and the smoking epidemic in particular, including: the worldwide dissemination of unhealthy behaviours like smoking brought about by trade liberalisation and multinational tobacco corporations; transnational anti-smoking advocacy networks made possible by Internet-based, communication technologies; and the adoption of global public health norms like those of the *FCTC*.

There is little doubt that temporalities and spatialities of globalisation shape many of the institutions, theories and practices that make up the contemporary field of global health and chronic disease. But, as I argued in the article, they are not the only notions of time and space at work within this field. Rather, they work alongside many other, often-contradictory spatio-temporal logics. The temporalities and spatialities underpinning the Lopez model explored in this article are a case in point. Unlike the temporalities and spatialities of sameness described in much of the literature on global health, the Lopez model is articulated around temporalities and spatialities of difference. This, of course, is not the difference celebrated by postmodern thinkers (e.g. Lyotard, 2004; Bauman, 2005), but the difference of modernisation theories built around nation–states, sequential stages and progress. Indeed, the Lopez model, in stark contrast to the ‘one world, one time, one health’ mantra of globalisation, divides the world into nation–states and orders them along epidemiological, geographical and development lines.

More generally perhaps, this article also speaks and contributes to the growing body of critical studies in global health (e.g. Fassin, 2012; Biehl and Petryna, 2013; Anderson, 2014). According to these scholars, the field of global health is characterised by ‘tensions and contradictions’ as well as ‘failures and resistances’ (Fassin, 2012, p. 107 and 113). They encourage us to recognise this ‘uneven terrain’ and think of ‘the globalisation of health … as a heterogeneous and contested historical phenomenon’ (Fassin, 2012, p. 99; Anderson, 2014, p. 372). This article very much follows this lead, depicting how different, even contradictory spatio-temporal logics, each with their own strengths and weaknesses, work alongside each other within the global health field. Furthermore, it shows the importance of knowledge and expert discourses such as globalisation and modernisation theories in the making of the notions and practices that are part of and inform contemporary global health, something also repeatedly emphasised in critical studies of global health (e.g. Lakoff and Collier, 2008; Reubi, 2013; Gaudillière, 2014).

## Footnotes

1 This research was articulated around three, main data collection strategies: (1) articles were gathered from a literature search on global tobacco control in major online databases and the British Library catalogue; (2) documents were obtained from organisations active in global tobacco control such as the WHO and the Framework Convention Alliance; and (3) over 100 semi-structured interviews were conducted with tobacco control experts and advocates in accordance with standard ethical principles and procedures (cf. Latour, 1993, Prainsack and Wahlberg, 2013). This corpus of texts and interviews was examined to identify the notions of temporality and spatiality around which the advocates and experts that make up the global tobacco control movement conceptualise, narrate and experience the smoking epidemic. Although these advocates and experts often come from different socio-economic and geographical backgrounds, it is important to note that their styles of thinking and reasoning are remarkably similar and consistent over time (Fleck, 1979, Hacking, 2002, Hass, 1992).

2 While the literature on globalisation and health has been very influential within the field of global health, it has existed alongside two other bodies of work. The first is the research on cost-effectiveness and the global burden of disease carried out by epidemiologists and economists like Dean Jamison, Christopher Murray and Alan Lopez. Concerned with health planning and financing in low- and middle-income countries, this work does not engage with theories of globalisation and is, like the Lopez model, markedly influenced by modernisation theories (e.g. Jamison et al., 1993, Murray and Lopez, 1996). The second is the anthropological and geographical research that is deeply sceptical of the global and celebrates the local, emphasising resistance to, mistranslation and re-appropriation of biomedical and public health discourses by community leaders, doctors and patients in particular places (e.g. Kelly and Beisel, 2011, Lawhon and Herrick, 2013, Livingstone, 2012).

3 As science and technology studies have suggested (e.g. Evans, 1999, Morgan and Morrison, 1999, Sismondo, 1999), scientific models like the epidemiological model devised by Lopez and his colleagues are best understood as heterogeneous assemblages of scientific theory, mathematical techniques, moral values, social categories, graphic representations, empirical data and narratives. At once representations, predictions and tools for intervention, they are critical in the production of contemporary scientific and political truths.
